# A Web-Based Stress Management Intervention for University Students in Indonesia (Rileks): Feasibility Study Using a Pretest-Posttest Design

**DOI:** 10.2196/37278

**Published:** 2022-07-19

**Authors:** Dilfa Juniar, Wouter van Ballegooijen, Mieke Schulte, Anneke van Schaik, Jan Passchier, Elena Heber, Dirk Lehr, Sawitri Supardi Sadarjoen, Heleen Riper

**Affiliations:** 1 Faculty of Psychology YARSI University Jakarta Indonesia; 2 Department of Clinical, Neuro and Developmental Psychology Vrije Universiteit Amsterdam Netherlands; 3 Department of Psychiatry Amsterdam University Medical Centers Amsterdam Netherlands; 4 Geestelijke Gezondheids Zorg inGeest Specialized Mental Health Care Amsterdam Netherlands; 5 HelloBetter – GET.ON Institut für Online Gesundheitstrainings GmbH Hamburg Germany; 6 Institute of Psychology Leuphana University Lüneburg Germany

**Keywords:** Indonesia, cultural adaptation, feasibility study, internet intervention, telemental health, digital mental health, low- and middle-income countries (LMIC), stress management, university students

## Abstract

**Background:**

University students are susceptible to excessive stress. A web-based stress management intervention holds promise to improve stress but is still at a novel stage in Indonesia.

**Objective:**

The aim of this paper was to report the feasibility of the intervention we developed—Rileks—among university students in Indonesia in terms of acceptability and usability, and to propose recommendations for future improvements.

**Methods:**

A single-group pretest and posttest design was used. Participants with scores of 15 or higher on the stress subscale of the 42-item Depression Anxiety Stress Scales were given access to the intervention (N=68). The main outcome measures were the 8-item Client Satisfaction Questionnaire (CSQ-8) score, the System Usability Scale (SUS) score, and intervention uptake. Participants’ experience in each session was evaluated using closed- and open-ended questions for future improvements. Descriptive statistics were used to examine primary outcome and qualitative session evaluations. Participants’ responses to each topic of the open questions were summarized.

**Results:**

The intervention was evaluated as being satisfactory (CSQ-8 mean score 21.89, SD 8.72; range 8-32). However, the intervention’s usability was still below expectation (SUS mean score 62.8, SD 14.74; range 0-100). The core modules were completed by 10 out of 68 participants (15%), and the study dropout rate was 63% (43/68) at postassessment. In general, the module content was rated positively, with some notes for improvement covering content and technical aspects.

**Conclusions:**

This study indicates that Rileks is potentially feasible for Indonesian university students. In order to be optimally applied in such a context and before scaling up web-based interventions in Indonesia, in general, further development and refinement are needed.

**International Registered Report Identifier (IRRID):**

RR2-10.2196/11493

## Introduction

Globally, an increasing number of university students experience stress [1-3]. To a certain extent, stress can be advantageous in stimulating human thriving [4]. However, ongoing high levels of stress may lead to negative outcomes, such as psychological distress, anxiety, depression, physical illness, substance abuse, and impaired work-related and academic performance [4-6]. The prevalence varies across studies and among countries, but overall, studies suggest that 20% to 25% of university students around the globe from various fields of study [7] suffer from psychological distress [3]. However, most of them do not receive support in reducing their high stress levels [5,8]. This is due to various reasons, including fear of stigma for seeking help for mental health problems [5] and limited availability of skilled mental health professionals within universities [5,9]. This has also been reported by university students in Indonesia [10], despite considerable need for psychological support (eg, two-thirds of Indonesian nursing and medical students experience moderate to severe levels of stress) [11,12].

Web-based interventions may overcome some of the issues related to this treatment gap. They may provide an accessible and potentially less stigmatizing alternative compared with face-to-face treatment, because clients can use web-based interventions privately [13,14]. Studies have shown that a web-based intervention can be a stand-alone intervention or can be an adjunct in a psychological intervention, which is known as a blended strategy [15,16]. A blended strategy offers some benefits, such as increasing an intervention’s acceptability [17], lowering dropout rates, and increasing the clinician’s efficiency [16]. However, implementing a blended strategy requires an adequate infrastructure and a mental health system that promotes the use of telemental health. In Indonesia, where an internet- or web-based intervention is still a novel approach and mental health service is still limited, a blended strategy might require more time to develop. Thus, as an initial step, we focused on a web-based intervention, as this would be more suitable for the current circumstances in Indonesia. Web-based interventions could be especially suitable for a university student population [18], since younger, well-educated individuals already tend to seek information and help for emotional and mental health problems through the internet [19-21]. A meta-analysis reported that web-based and computer-delivered stress management interventions can effectively diminish university students’ stress, with an effect size of 0.73 (95% CI –1.27 to –0.19, *P*=.008) [22]. A web-based stress management intervention culturally adapted for Indonesian university students might be feasible because of the increasing availability of the internet in Indonesia [23]; consequently, there would be greater internet access at their universities and, to a lesser extent, at home.

Several web-based stress management interventions for university students have been developed in high-income countries [22]. GET.ON Stress has been investigated in several randomized controlled trials [24-26], is based on the transactional model by Lazarus and Folkman [27], and consists of problem-focused and emotion-focused coping strategies. It was originally developed for German employees, has been adapted for German-speaking university students, and has been renamed StudiCare Stress [28]. As part of this project, the GET.ON Stress intervention was culturally adapted to the Indonesian context, using the integrative cultural adaptation model by Barrera et al [29] as a guideline. The first two steps of the adaptation process, which have been previously reported [30], have led to the Rileks intervention; Rileks means relax, and means calm in the Indonesian language.

Due to the novelty of this kind of intervention in Indonesia, instead of a pilot study, a feasibility study was conducted as the third step with an emphasis on the process of developing, implementing, and assessing preliminary responses of participants to the new intervention [31,32]. Furthermore, a feasibility study helps to evaluate components (ie, participant recruitment, accuracy of the intervention protocol, and ability to execute the new intervention) necessary for the next large-scale study [33,34]. This paper reports on the third step of the model by Barrera et al [29], which is the feasibility study of the preliminary version of Rileks.

The primary aim of this study was to investigate the feasibility of Rileks among university students in Indonesia in terms of acceptability, usability, and intervention uptake. The secondary aim was to investigate stress, anxiety, and depression reduction and improvement of quality of life, as well as to generate feedback for further refinement of Rileks.

## Methods

### Participants and Sample Size

Inclusion criteria were for participants to have scored 15 (ie, low stress level) or higher on the 42-item Depression Anxiety Stress Scales (DASS-42) [32], to be enrolled in a university in Indonesia, to be 19 years of age or older, to have access to the internet, and to be able to speak Bahasa Indonesia fluently. Participants were recruited between October 10 and 19, 2018. Information about our study and website was disseminated through social media platforms, such as Facebook, Instagram, and WhatsApp groups, and through presentations by the principal investigator (DJ) at events at two universities in Indonesia: YARSI University and the Indonesian State College of Accountancy.

A formal calculation of sample size used for effectiveness trials is not suitable for a feasibility study [35]. In our study, we intended to include at least 50 participants, with a saturation of 75 participants to ensure sufficiently reliable estimates of our main study parameters. We based this estimate on a systematic study analyzing sample sizes in pilot and feasibility studies in the United Kingdom, which reported a median of 36 participants for a feasibility study sample size [35].

### Study Design and Procedure

A single-group pretest and posttest design was used. Interested university students who signed up on our website subsequently received a link to the screening measurement, the DASS-42 stress scale. Eligible university students then had to submit their signed electronic informed consent form before completing the baseline measurements (ie, pretest). All included participants received log-in credentials for the intervention, and only those who logged in received posttreatment measurements (ie, posttest) 10 weeks after the pretest. A duration of 10 weeks was chosen as the postmeasurement time point, as we considered this to be sufficient time for participants to complete the intervention. All measures were self-reported and administered online.

### Ethics Approval

The study was reviewed and approved by the Indonesian ethics committee at YARSI University (project No. 193/KEP-UY/BIA/VIII/2017).

### Intervention

Rileks consists of six sessions and an optional booster session provided 4 weeks after intervention completion. The first session comprises psychoeducational information about stress, based on the emotion-focused and problem-focused coping strategies by Lazarus and Folkman [27]. The second session comprises the six-step problem-solving method based on problem-solving therapy [36,37]. In the second session, participants work on their problem-solving skills by applying the method to their individual problem. In sessions 3 to 5, participants are introduced to emotional regulation techniques based on affect regulation training [37,38]. The techniques include muscle and breathing relaxation, acceptance and tolerance of emotions, and self-support in difficult situations. These techniques are explained one by one in each session, respectively. In the last session, participants are asked to reassess their goals for the training and to identify their personal warning signs for stress. Furthermore, participants are asked to write a letter to themselves about how they imagined their life would be after applying the methods and techniques they had been taught. In addition, a booster session may be given as an option to evaluate the letter they had written to themselves in the last session, reassess their goals, and make plans to continue applying what they had learned from Rileks.

Each session contains general information, examples related to the exercises, exercises guided by electronic coaches (e-coaches), quizzes, slideshows encompassing explanations related to stress management methods and techniques, audio files, and downloadable material, which were all presented on a secure platform. Access to the platform was given to the participants based on their email addresses and self-designated passwords. Participants were advised to log in once or twice per week. A reminder was sent to the participants if they did not log in within 7 days. Within 48 hours after completion of each session, four trained psychologists acting as e-coaches gave personalized written feedback on the exercises. The e-coaches followed guidelines about the feedback process that are defined according to the standardized manual on feedback writing for the intervention.

### Primary Outcome Measures

Acceptability was measured by assessing clients’ satisfaction with Rileks using the translated version of the 8-item Client Satisfaction Questionnaire (CSQ-8) [39-41]. The scale consists of eight questions answered using 4-point Likert scales (scored from 1 to 4), with total scores ranging from 8 (“great dissatisfaction”) to 32 (“great satisfaction”). We set an average score of above 20 (20 is the median total score) as the criterion for acceptable satisfaction. The CSQ-8 has good reliability, as it has been reported to have a Cronbach α of .92 [42].

The Indonesian version of the System Usability Scale (SUS) [43] was used to assess the usability of Rileks in terms of user friendliness [44,45]. The scale comprises 10 statements scored on a 5-point Likert scale, ranging from 1 (“strongly disagree”) to 5 (“strongly agree”). The total scores were then transformed into a scale with scores ranging from 0 to 100, with higher scores representing higher usability. A score of 70 or more was considered adequate as a feasibility criterion [45]. The Cronbach α of the Indonesian version has been reported as .84 [43], which indicates good reliability. Both the CSQ-8 and the SUS were only administered at postintervention.

Intervention uptake was measured by assessing the number of participants who completed the core online sessions (ie, sessions 1 to 5), where participants learn the basic principles of problem solving and emotion regulation. The criterion for acceptable adherence was set at 60% or more participants who completed the core sessions [30]. The 60% threshold was based on previous meta-analyses on adherence to internet-based cognitive behavioral therapy (CBT) for depression, which found that 65.1% of participants completed the entire internet-based CBT sessions [46].

### Secondary Outcome Measures

The severities of stress, anxiety, and depression were measured using the Indonesian version of the DASS-42 [47]. The scale consists of 42 items divided into three subscales—depression, anxiety, and stress—where each subscale contains 14 items ranging from 0 to 3 and a higher score indicates a greater degree of severity [48]. The Indonesian version of the DASS-42 shows excellent overall reliability, with a Cronbach α of .95, and high internal consistency in the separate depression, anxiety, and stress subscales (α=.91, .85, and .88, respectively) [47].

Quality of life was measured by the Indonesian version of the brief version of the World Health Organization Quality of Life instrument (WHOQOL-BREF) [49]. It consists of two items that measure overall quality of life and general health, and 24 items that measure how the respondent felt in the last 2 weeks across four domains: physical health, psychological health, social relationships, and environmental health [49,50]. Among the Indonesian population, the WHOQOL-BREF has been reported as having internal consistencies, or Cronbach α values, of .41 to .77 in the four domains, respectively [51], and reliability with intraclass correlation coefficients of 0.70 to 0.79 in the four domains, respectively [49]. The DASS-42 and the WHOQOL-BREF were assessed both at pretest and posttest.

### Other Measurements

Demographic variables were collected to describe the characteristics of the study population. Cultural-related aspects of the Rileks modules were evaluated within the aspects of language, case examples, and visual presentation. With regard to language use, we asked whether the participants could understand the words in each session. To evaluate case examples and visual presentation, the participants were asked whether the case examples and pictures used in the sessions represented the Indonesian university student context. Participants’ experience with e-coaches was evaluated using six questions concerning participants’ satisfaction with contact with their e-coaches. Some of the questions were adapted from another study that assessed participants’ satisfaction with online therapeutic contact [52]. A selection of responses was provided for each question. An open question asked for participants’ recommendations for future improvement of the e-coaches.

Furthermore, by the end of each session on the intervention platform, participants gave an evaluation about their experience in each session. The participants assessed general aspects of each session (eg, usefulness, easiness, and time needed to complete the module) using a Likert scale ranging from 1 (eg, “very useful” or “very easy”) to 5 (eg, “not useful at all” or “very difficult”). They also assessed specific aspects of the sessions (eg, the structure of the module) using a Likert scale ranging from 1 (“positive judgement”) to 7 (“negative judgement”) and answered some open questions (eg, “What did you like and not like about this session?” and “How might you have benefitted from it?”).

### Statistical Analyses

Descriptive statistics were used to examine primary outcomes, demographic data, and session evaluations. Participants’ satisfaction, using the CSQ-8, and system usability, using the SUS, as feasibility parameters were summarized using means and SDs. Intervention uptake was summarized with frequency of participants who logged in or completed each session. Differences in demographic characteristics between participants who logged in and those who did not were tested using a chi-square test in terms of sex, level of education, field of study, and university location. Independent-sample *t* tests were used to assess differences in mean age, DASS-42 scores, and WHOQOL-BREF scores.

Secondary outcomes were analyzed using 2-tailed paired-samples *t* tests with a level of significance of *P*=.05. A normality test at each time point was conducted beforehand, using the Shapiro-Wilk test. As the distributions significantly differed from normal (*P*<.05), a sensitivity analysis using nonparametric tests (eg, the Wilcoxon signed-rank test) was conducted. Furthermore, within-group effect sizes (Cohen *d*) were calculated. SPSS software (version 26; IBM Corp) was used for the analysis.

In order to understand the participants’ experience and provide recommendations relevant to future refinement, participants’ responses to the open questions for each topic were summarized by categorizing responses with similar themes. Categorization was done by the principal investigator (DJ) and one of the Indonesian team members as a second rater. The summary was finalized by consensus between the two.

## Results

### Enrollment and Participant Characteristics

**Figure 1:**
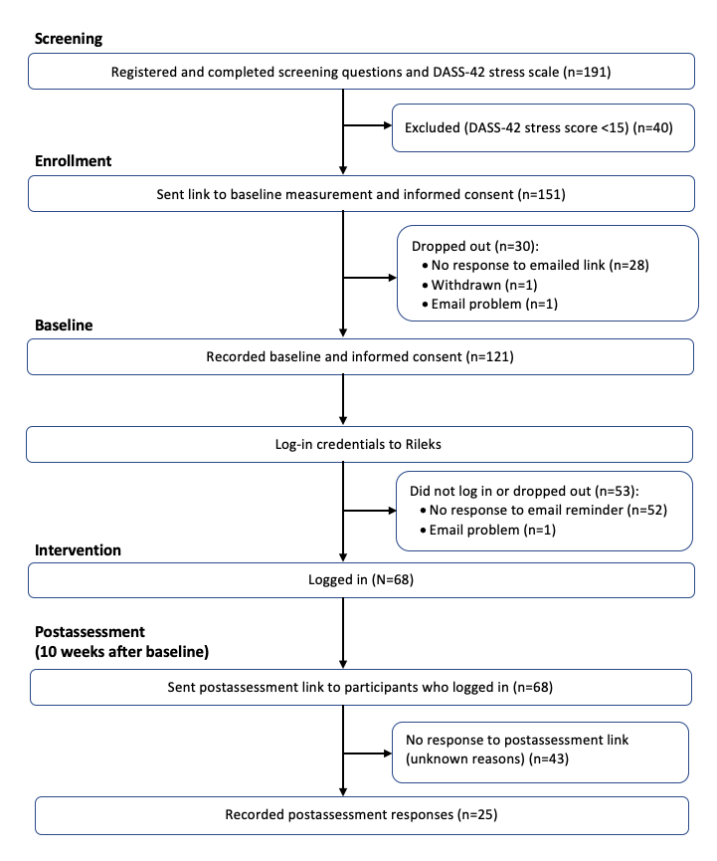
Study flowchart.

A total of 191 university students registered and completed the screening on the study website. Most of the participants (n=169, 88.5%) learned about the study through social media (eg, Facebook, Instagram, and WhatsApp groups). A small proportion (n=22, 11.5%) were informed through presentations by the principal investigator (DJ) at two universities. Of the 191 registered university students, 40 (20.9%) scored lower than 15 on the DASS-42 stress scale, which can be considered as normal stress, and 151 (79.1%) were eligible for inclusion. See Figure 1 for study flowchart.

Baseline questionnaires were completed by 121 out of 151 (80.1%) participants. The majority were female (n=103, 85.1%), had a bachelor’s degree (n=94, 77.7%) in social and behavioral sciences (n=83, 68.6%), and studied on the island of Java (n=79, 65.3%). The average DASS-42 stress score at baseline was 23.11 (SD 5.8), indicating moderate stress. One-third of the participants (n=41, 33.9%) fell into the severe stress category (ie, score 26-33). Those who logged in mostly studied in Java and scored lower on psychological quality of life compared to those who did not log in. See Table 1 for all participants’ characteristics at baseline.

**Table 1:** Characteristics of participants at baseline and comparisons between those who did not log in and those who did.

Characteristic		Baseline (n=121)	Participants who did not log in (n=53)	Participants who logged in (N=68)	*P* value^a^
**Age (years)**					
	Range	19-42	19-39	19-42	N/A^b^
	Mean (SD)	24.03 (4.61)	24.7 (4.41)	23.56 (3.55)	.12
**Sex, n (%)**					.49
	Female	103 (85.1)	44 (83.0)	59 (86.8)	
	Male	17 (14.0)	9 (17.0)	8 (11.8)	
	Did not fill out	1 (0.8)	0 (0)	1 (1.5)	
**Level of education, n (%)**					.24
	Bachelor’s degree and equivalent	94 (77.7)	46 (86.8)	48 (70.6)	
	Master’s degree	18 (14.9)	5 (9.4)	13 (19.1)	
	Doctoral degree	3 (2.5)	1 (1.9)	2 (2.9)	
	Did not fill out	6 (5.0)	1 (1.9)	5 (7.4)	
**Field of study, n (%)**					.29
	Social and behavioral science	83 (68.6)	30 (56.6)	53 (77.9)	
	Business and administration	12 (9.9)	9 (17.0)	2 (2.9)	
	Languages	6 (5.0)	4 (7.5)	0 (0)	
	Humanities	0 (0)	3 (5.7)	0 (0)	
	Others	20 (16.5)	7 (13.2)	13 (19.1)	
**University location, n (%)**					.04
	Java	79 (65.3)	30 (56.6)	49 (72.1)	
	Sumatra	20 (16.5)	10 (18.9)	10 (14.7)	
	Kalimantan or Borneo	8 (6.6)	2 (3.7)	6 (8.8)	
	Sulawesi	10 (8.3)	8 (15.1)	2 (2.9)	
	East Nusa Tenggara	2 (1.7)	2 (3.8)	0 (0)	
	Others	2 (1.7)	1 (1.9)	1 (1.5)	
**DASS-42^c^ subscale score, mean (SD)**					
	Depression	14.88 (8.70)	13.37 (8.21)	16.16 (8.90)	.92
	Anxiety	15.54 (6.72)	15.54 (6.92)	15.58 (6.24)	.99
	Stress	23.11 (5.80)	22.89 (5.91)	23.28 (5.53)	.71
**Stress level, n (%)**					.24
	Mild	36 (29.8)	16 (30.2)	20 (29.4)	
	Moderate	40 (33.1)	16 (30.2)	24 (35.3)	
	Severe	41 (33.9)	20 (37.7)	21 (30.9)	
	Extremely severe	4 (3.3)	1 (1.9)	3 (4.4)	
**WHOQOL-BREF^d^ domain score, mean (SD)**					
	Physical health	41.83 (7.49)	42.15 (7.23)	41.56 (7.82)	.56
	Psychological health	47.90 (14.29)	51 (14.07)	45.34 (13.98)	.02
	Social relationship	49.04 (19.29)	48.69 (20.65)	49.33 (18.39)	.43
	Environmental health	53.91 (11.67)	53.71 (11.97)	54.07 (11.61)	.98
	Overall quality of life	2.98 (0.87)	3.08 (0.88)	2.91 (0.86)	.29
	Overall health	2.90 (0.89)	3.04 (0.79)	2.79 (0.97)	.15

^a^*P* values are based on the difference between those who logged in and those who did not.

^b^N/A: not applicable; the *P* value was not calculated for these values.

^c^DASS-42: 42-item Depression Anxiety Stress Scales; scores range from 0 to 42 for each subscale, where a higher score indicates increased severity.

^d^WHOQOL-BREF: brief version of the World Health Organization Quality of Life instrument. Scores range from 1 to 5 for each item in each domain; the total score for each domain was then transformed linearly to a scale ranging from 0 to 100, where a higher score indicates increased quality of life.

### Primary Outcomes

A total of 10 weeks after the pretest, posttreatment questionnaires were sent to the 68 participants who had logged in. Questionnaires were completed by 25 participants; thus, the study dropout rate was 63% (43/68). The CSQ-8 mean score was 21.89 (SD 8.72), which met our acceptable satisfaction criterion of a mean score of above 20. Table 2 provides the mean scores of the CSQ-8 items.

**Table 2:** Scores for all items of the 8-item Client Satisfaction Questionnaire for Rileks (n=25).

Item	Score, mean (SD)^a^
How would you rate the quality of service you have received from Rileks?	3.20 (0.71)
Did you get the kind of service you wanted?	2.92 (0.70)
To what extent has Rileks met your needs?	2.72 (0.61)
If a friend were in need of similar help, would you recommend Rileks to him or her?	3.24 (0.59)
How satisfied are you with the amount of messages you have received from Rileks?	3.16 (0.55)
Has Rileks helped you to deal more effectively with your problems?	3.28 (0.74)
In an overall general sense, how satisfied are you with the help you have received from Rileks?	2.96 (0.73)
If you were to seek help again, would you use Rileks again?	3.04 (0.68)

^a^Items were rated on a 4-point Likert scale, ranging from 1 to 4, where higher scores indicate increased satisfaction.

The SUS mean score was 62.80 (SD 14.74), with the lowest score for the learnability item (Table 3). With regard to intervention uptake, all 121 enrolled participants received log-in credentials for Rileks by email. Of those participants who enrolled, 68 (56.2%) logged in. These 68 participants did not differ from those who never logged in (n=53, 43.8%) on any of the baseline characteristics, with the exception of university location (*χ*^2^_26_=21.2, *P*=.04) and psychological quality of life (t_113_=2.14, *P*=.02; Table 1). Of the 68 participants who logged in, the core sessions were completed by 10 (15%) participants. This number is below our acceptable uptake criterion of 60%. Reasons for nonadherence were mostly unknown because those participants could not be reached. The known reasons for nonadherence or withdrawal included time management problems, rare use of email so they missed notifications, unexpected events (eg, internship to a remote village with limited internet coverage), and technical problems, such as unfamiliarity with the log-in system. Table 4 outlines the number of completed sessions in Rileks.

**Table 3:** Scores for all items of the System Usability Scale for Rileks (n=25).

Item	Score, mean (SD)^a^
I think that I would like to use Rileks frequently.	3.48 (0.82)
I found Rileks unnecessarily complex.^b^	3.40 (1.04)
I thought Rileks was easy to use.	3.56 (0.96)
I think that I would need the support of a technical person to be able to use Rileks.^b^	3.88 (1.01)
I found that the various functions in Rileks were well integrated.	3.72 (0.89)
I thought there was too much inconsistency in Rileks.^b^	3.56 (0.77)
I would imagine that most people would learn to use Rileks very quickly.	3.88 (0.73)
I found Rileks very cumbersome to use.^b^	3.44 (1.01)
I felt very confident using Rileks.	3.32 (0.85)
I needed to learn a lot of things before I could get going with Rileks.^b^	2.88 (1.27)

^a^Items were rated on a 5-point Likert scale, ranging from 1 to 5, where higher scores indicate increased usability.

^b^The scores for this item were reversed.

**Table 4:** Rileks sessions completed by participants.

Step	Participants (N=68), n (%)
Logged in	68 (100)
Completed module 1	40 (59)
Completed module 2	26 (38)
Completed module 3	16 (24)
Completed module 4	12 (18)
Completed module 5	10 (15)
Completed all 6 modules	9 (13)

### Secondary Outcomes

At posttest, out of 25 participants who completed the CSQ-8 and the SUS, the DASS-42 stress scale and the WHOQOL-BREF were completed by 23 (92%) participants, consisting of the core session completers and noncompleters. At posttest, participants reported significantly lower levels of stress (mean –10.04, 95% CI 5.36-14.72), depression (mean –6.85, 95% CI 1.37-12.32), and anxiety (mean –6.45, 95% CI 1.56-11.34), with a high effect size on stress and moderate effect sizes on anxiety and depression. Participants also reported significantly improved quality of life in terms of physical health (mean –21.25, 95% CI –29.05 to –13.44), psychological health (mean –12.50, 95% CI –20.25 to –4.75), overall quality of life (mean –0.55, 95% CI –0.96 to –0.13), and overall health (mean –0.70, 95% CI –1.31 to –0.09). However, we did not find significant differences in the social relationship and environmental health aspects of quality of life (Table 5). Wilcoxon signed-rank tests for nonparametric distributions yielded the same results.

**Table 5:** DASS-42 and WHOQOL-BREF scores at baseline and posttreatment (n=23).

Measure			Baseline score, mean (SD)	Postassessment score, mean (SD)	*P* value	Cohen *d*
**DASS-42^a^ subscales**						
		Stress	24.74 (6.33)	14.70 (11.41)	<.001	0.93
		Anxiety	17.95 (8.35)	11.50 (8.34)	.01	0.62
		Depression	17.10 (10.74)	10.25 (11.39)	.02	0.58
**WHOQOL-BREF^b^ domains**						
	Physical health		42.30 (8.28)	63.55 (13.95)	<.001	1.25
	Psychological health		43.85 (17.74)	56.35 (20.14)	.003	0.78
	Social relationship		49.40 (17.99)	54.35 (19.64)	.45	0.19
	Environmental health		54.55 (13.32)	50.60 (15.14)	.13	0.41
	Overall quality of life		2.70 (1.08)	3.25 (1.07)	.01	0.62
	Overall health		2.70 (1.03)	3.40 (0.94)	.03	0.54

^a^DASS-42: 42-item Depression Anxiety Stress Scales; scores range from 0 to 42 for each subscale, where a higher score indicates increased severity.

^b^WHOQOL-BREF: brief version of the World Health Organization Quality of Life instrument. Scores range from 1 to 5 for each item in each domain; the total score for each domain was then transformed linearly to a scale ranging from 0 to 100, where a higher score indicates increased quality of life.

### Feedback for Future Refinement

#### Module and Session Evaluation

In general, participants rated the individual modules positively (Table 6). The response summary of the open questions (Multimedia Appendix 1) indicated that, in general, participants were in favor of the clear examples given in the modules and the case examples, to which they felt they could relate. Furthermore, participants suggested that the module content had provided them with new and comprehensive information as well as exercises that could help them manage their stress in terms of recognition, being more reflective and accepting of oneself, learning how to examine problems, and thinking of positive activities that could help them manage their stress. Moreover, Rileks was considered to be a medium where participants were able to express their problems openly without feeling ashamed.

Participants also mentioned things they did not like, mostly technical problems, such as unfamiliarity with the system, poor audio quality, and some files being too large to download, as well as some repeated questions and confusion about how to do the exercises, despite the given examples.

**Table 6:** Module evaluation.

Evaluation criteria	Evaluations by module					
	Module 1 (n=40)	Module 2 (n=26)	Module 3 (n=16)	Module 4 (n=12)	Module 5 (n=10)	Module 6 (n=9)
General usefulness	Useful	Useful	Useful	Very useful	Useful	Very useful
Easy to complete	Easy	Easy	Easy	Easy	Easy	Very easy
How long did it take? (minutes)	30-60	30-60	30-60	30-60	60-90	30-60
Clarity	Undecided	Clear	Clear	Clear	Clear	Very clear
Subcontent usefulness	Undecided	Useful	Useful	Useful	Useful	Very useful
Pleasantness	Undecided	Pleasant	Undecided	Pleasant	Pleasant	Very pleasant
Comprehensibility	Undecided	Comprehensible	Comprehensible	Comprehensible	Comprehensible	Very comprehensible

Suggestions for improvements included the following: adding interactive functions for communication with the e-coach or other professionals to get direct help, such as live chat, especially in parts where participants need to remember and deal with negative events they had; making the intervention simpler and shorter; adding links to music and video guidance, especially for relaxation; providing downloadable materials; and developing a better display for those accessing Rileks from a mobile phone.

#### Cultural and Technical Aspects

At posttest, all participants (n=23) could understand the language used in the Rileks modules, including idioms and metaphors. Most participants (n=20, 87%) thought that Rileks used suitable media, such as audio guidance for relaxation or a slide show to explain theory. Case examples in Rileks were considered to represent the Indonesian university student context (n=22, 96%). Furthermore, one participant suggested adding a case example that speaks for university students who come from a family with a low economic background.

#### e-Coach Evaluation

Of the 23 participants at posttest, 13 (57%) said they missed face-to-face communication and 7 (30%) had hoped for more support. The quality of support was generally considered positive. The e-coach support helped participants gain insight into managing their problems and go through the modules with confidence and motivation. Moreover, the presence of e-coaches made them feel understood and less lonely.

Open questions about suggestions for future refinement were responded to by 22 (96%) participants. Suggestions for refinement from the open questions can be summarized as follows. Firstly, the feedback process could be more interactive and personal (n=3, 13%). Participants hoped that they would be able to ask further questions by replying to the feedback they had received. Secondly, some participants suggested the use of a medium other than email to deliver feedback (eg, using chat; n=2, 9%). Thirdly, they would like the possibility of maintaining a future connection with the e-coaches for further counseling after the intervention had ended (n=2, 9%). Lastly, there was a suggestion to combine offline and online treatment to meet the needs of a participant who felt that they could not fully express themselves to the e-coach through written text (n=1, 4%).

## Discussion

### Principal Findings

The main aim of this study was to evaluate the feasibility of Rileks as part of an adaptation process of a web-based stress management intervention among university students in Indonesia. Rileks was reported as being acceptable, even though its usability and intervention uptake were still below our expected criteria levels. Study findings showed that participants’ stress level and quality of life improved at posttest. The intervention was appreciated by participants in terms of content usefulness, easiness to complete, comprehensibility, suitability for the Indonesian university student context, and the quality of e-coach support, with more e-coach interaction being desired.

### Primary Outcomes

Rileks was rated as generally satisfactory, which indicates that the intervention was acceptable. The most satisfying aspect of Rileks as perceived by the participants was that the intervention helped them deal with their problems effectively and that they would recommend it to their friends. With regard to usability, Rileks received a lower than expected threshold score, with the learnability aspect as a main challenge. As reported, participants needed to learn and become familiar with a number of new technical aspects related to the system before they could engage with the intervention. We considered this to be due to the fact that a web-based intervention was relatively new in Indonesia, hence, participants did not have much prior experience in using such an intervention, which may have affected their perception of its usability [53]. Another possible explanation is that the user interface of the system is not user friendly enough. Even though learnability was a challenge, participants felt positive about being able to learn it in a relatively short period of time.

Usability issues were also found in internet-delivered mental health treatments in developed countries [54]. While usability is an essential part of system development, assessing usability of a web-based intervention system is still challenging. As usability is closely connected with interaction design, we are challenged with human-computer interaction issues that are still poorly understood. Furthermore, studies also revealed the need for a guideline for testing the usability of internet-delivered treatment systems [54] and a standardized usability questionnaire for such interventions [55].

The study findings revealed that the uptake of the intervention’s core modules was below our criterion level. However, the number of participants who completed the core modules in our study still fell within the range of the reported number of module completers in a systematic review on computerized CBT (12%-100%) [56]. Moreover, a systematic review reported that only 30% of patients adhered to treatment until the third session of face-to-face interventions [57], making an uptake of 24% for module 3 sufficient, considering that computerized CBT is more likely to have a lower adherence rate [56]. Thus, we consider that Rileks still has potential, even though it did not yet meet the uptake criterion level.

Compared to the GET.ON Stress and StudiCare Stress interventions, Rileks’ adherence rate of 15% for its core modules still falls behind. Studies of the GET.ON Stress intervention revealed an adherence rate range of 41.9% to 71.8% for its modules [24-26,58,59]. While the StudiCare Stress adherence rate was reported to be high, on average, the participants completed 74.7% of the intervention [60]. The findings seem in line with participants’ overall satisfaction with the intervention. Based on the CSQ-8, the StudiCare Stress intervention had a very high satisfaction rate [60], and the GET.ON Stress intervention had high to very high satisfaction rates in its studies [24-26]. Rileks itself had an acceptable overall satisfaction level. Participants perceived that the intervention had helped them to deal more effectively with their problems, but they still considered Rileks to be suboptimal in meeting their needs or to be the kind of service they wanted.

A few reported reasons for nonadherence included time management issues (eg, being too busy), having competing activities, and technical problems, such as unfamiliarity with the log-in system. This is supported by a systematic review that argued that unfamiliarity with computers or the internet and feeling too busy to complete treatment may contribute to dropout from internet-based treatment [61,62]. Other reasons for intervention dropout in our study were that participants missed notifications because they seldom used or checked their email. This implies the need for future studies to use means other than email to send reminders to participants in order to boost intervention uptake, such as personal messages via preferred platforms (eg, instant chat messaging). Furthermore, problems with internet connections in rural areas were also experienced, which indicate that other forms of communication or channels, such as a mobile phone apps, may also be explored as potential options, as the use of mobile apps does not always require constant internet connectivity.

Furthermore, nearly 50% of participants dropped out by the end of module 1, which was the largest number of dropouts compared to other modules. Among all modules, module 1 also received the most “undecided” responses in the evaluation categories of clarity, subcontent usefulness, pleasantness, and comprehensibility. This result may be due to several possibilities. One possibility is that those who dropped out after module 1 were interested in and curious about the intervention at first, but found out that a module-based intervention was not suitable for them after finishing module 1. Another possibility is that participants’ experience was suboptimal during module 1 due to a delivery method of psychoeducational content that was less than ideal for university students. This is in line with a systematic review that reported that young people tend to perceive educational material as unengaging, which caused dropout [63]. Further investigation on how to deliver psychoeducational material in engaging ways for university students in Indonesia is needed for future refinement.

Interestingly, it was found that stress severity level was associated with adherence, as those who logged in and engaged tended to have higher stress levels and significantly poorer psychological quality of life compared to those who did not log in. This is supported by other studies that confirmed that participants with less severe problems and difficulties may be less motivated and are subsequently more likely to drop out of internet-based treatment [61,64].

### Secondary Outcomes

Participants’ stress levels at the posttreatment assessment were significantly lower than at the pretreatment assessment. In addition, the levels of anxiety and depression were also significantly lower among participants at postintervention. This finding is in line with a previous meta-analysis that reported that face-to-face and internet-based interventions do reduce stress levels as well as symptoms of anxiety and depression among university students [22,65,66]. Furthermore, participants’ overall quality of life as well as their health and psychological quality of life were better at posttreatment compared with pretreatment. Our study findings may give initial implications for the clinical impact of Rileks, but should be interpreted with caution due to our study design and small sample size.

### Future Web-Based Intervention Studies and Rileks Refinements

Our findings demonstrate that web-based intervention studies targeting university students in Indonesia may be feasible. We reached 191 potential participants from several islands in Indonesia in 10 days, with 80.1% (121/151) of invited participants giving their consent and filling in baseline questionnaires. This indicates that university students in Indonesia were interested in our study. The use of the internet, social media, and particularly WhatsApp groups play an important role in the process of reaching targeted potential participants. These strategies allowed us to reach potential participants from many different cities, islands, and fields of study with relatively little effort. Furthermore, taking part in activities involving the target group was also useful in disseminating information on the study. Such activities allowed face-to-face interaction between the principal investigator (DJ) and the target group, where the principal investigator could give information on stress and our study, thus increasing university students’ awareness of stress and the credibility of our study. However, our study had a relatively high dropout rate (63%), which is commonly found in other internet-based interventions [56]. It is unfortunate that we did not have sufficient data to explain the reasons for dropout due to the unresponsiveness of our participants. This may be because we approached the participants who dropped out via email, which was not the most convenient medium for them. Thus, for future studies, we recommend using other ways to approach study participants (eg, sending a short survey about reasons for dropout through any of their preferred devices or platforms).

Participants’ feedback for improving future versions of Rileks related to both content (eg, scope of case examples and wording) and technical aspects (eg, suggesting a medium other than email to send notifications and a better display on the mobile phone version). Involvement of relevant stakeholders in the process of refining the content and technical aspects would be valuable for the purpose of achieving an optimal form of Rileks. One highlight of our findings was the request for the availability of interactive communication with the e-coach. This suggested that even though university students in Indonesia are open to the use of internet-based interventions, face-to-face or interactive communication is still preferred. This outcome is in line with other research that suggests that face-to-face interaction is still considered an essential and significant element of mental health services [64,67,68]. According to the latter study, Indonesian people in general would still prefer face-to-face contact, especially when they can access it nearby. This condition may make the use of a web-based intervention to overcome the mental health gap in Indonesia seem challenging. One alternative is to use lay counselors (eg, psychology students trained as e-coaches). Furthermore, reaching out and involving local community health centers to support interventions may be a possible means for future dissemination and implementation of web-based interventions in Indonesia [69]. Another alternative is using a blended strategy by combining modules and instant chat or video conference for more interactive communication between the client and the e-coach.

Our findings indicate that a web-based psychological intervention such as Rileks is acceptable among Indonesian university students and has the potential benefit of clinical effectiveness. Thus, it may provide a good opportunity for university students to have psychological support when there is a mental health service gap within universities and stigma for seeking mental health care. A refinement incorporating all input from participants to overcome challenges of usability and dropout rates is needed. In order to incorporate all input related to the content, technical aspects, and e-coach support into the intervention, involvement and collaboration of relevant stakeholders will be very important (eg, university students, clinicians, lay counselors, software developers, and user interface and user experience experts). Input from participants that we highlight from this study is their desire to have more interactive e-coach support. Further study should make sure that the amount and type of e-coach support that is needed throughout the intervention is provided (eg, chat, video call, email, time of support, and duration). Another shortcoming that we need to tackle is optimizing participants’ experience while they are going through the modules, especially the psychoeducational module. Future studies may experiment to find the most engaging way to deliver psychoeducational content to university students in Indonesia.

### Strengths and Limitations

To the best of our knowledge, Rileks is the first web-based intervention to provide stress management in the Indonesian university student context. Furthermore, it is among the first web-based psychological interventions being culturally adapted from Western culture to Asian culture. In addition, other than quantitative assessment, our study also considered qualitative data from open questions to give more insight into participants’ experience in working with the Rileks modules and system, including e-coaches. However, our study also has a number of limitations. The dropout rate from the enrollment stage to postassessment was relatively high. The data obtained at the postassessment only came from 37% of the participants who were logged in to the intervention and, thus, did not equally represent all participants. Furthermore, 85.1% of the participants were female and, thus, not likely to represent the real university student population, which consists of 56.1% female students and 43.9% male students [70].

### Conclusions

Rileks shows potential feasibility for Indonesian university students. However, our findings also underscore the need for further development of this kind of intervention in order to optimize the experience for Indonesian university students. Further refinements are needed regarding content and technical aspects. Despite the potential of web-based interventions and telemental health, in general, to minimize the mental health gap among the Indonesian population, our study implies that more work needs to be done before we can scale up this kind of intervention in Indonesia. In sum, the intervention has potential, but it needs refinement before it can be optimally applied in this setting.

## Appendix

Multimedia Appendix 1 Response summary of open questions on the evaluation of the modules.

## Abbreviations

CBT: cognitive behavioral therapy
CSQ-8: 8-item Client Satisfaction Questionnaire
DASS-42: 42-item Depression Anxiety Stress Scales
e-coach: electronic coach
SUS: System Usability Scale
WHOQOL-BREF: brief version of the World Health Organization Quality of Life instrument
