# Supplementary material for: A Web-Based Stress Management Intervention for University Students in Indonesia (Rileks): Feasibility Study Using a Pretest-Posttest Design
Source: JMIR Form Res. 2022 Jul 19;6(7):e37278. doi: 10.2196/37278 (PMC9491829; doi:10.2196/37278)
Supplement: Multimedia Appendix 1 [file formative_v6i7e37278_app1.pdf]

This is a Multimedia Appendix to a full manuscript published in the JMIR Formative Research. For full copyright and citation information see http://dx.doi.org/10.2196/jfr.37278

Response summary of open questions on module evaluation.

	Point of summary	Quotes
**What did you like about the sessions?**		
	New and useful information and knowledge	A lot of new information (P0104).<br>Basic knowledge related to stress (P032).<br>There were a lot of new information and cases on stress management that I could learn from (P0104).<br>Easy and very useful knowledge (P0108).<br>Could expand my insight (P0144).
	Relevant examples	There was audio and examples of people who experienced the things I experienced (P045).<br>Explanation accompanied with real examples (P059).<br>The introduction and the impact, the examples were also good (P070)<br>The example is very helpful (P0126).<br>There are examples of how to fill in the answer and the language is easy to understand. It was shown that there are other people who also feel the same like us (P0131).<br>There are sound recordings and examples (P0144)<br>The module is quite comprehensive with relevant examples (P085).<br>Everything had its explanation and followed by practice (P054).
	Helping me to contemplate on my problem	…Examine the problem (P0113).<br>Reveal the problems we have (P054).<br>Explain stress analysis and stress journals that are very useful even when all these interventions are completed (P088).<br>I can recognize my stress (P0133).<br>…to think more deeply related to the problem I was facing (P0123)
	Help to overcome my stress	I like it when I am thinking about activities that will be carried out for s tress management (P094).<br>I Just like to fill in the answer something that helps make happy and release my stress (P0127).<br>Motivate me to not become stress because there are already many people who succeed when doing it (P0147).<br>I like to be reminded about positive things that can help me to not stress (P033)

	Help to recognize oneself	Guidance for more self-reflection (P043)<br>Remind me of the things I like (P041).<br>Being able to recognize myself (P052).<br>……the sessions made me try to explore myself more deeply…. (P0123).
	Confide	Express opinions (P0148).<br>I can express what I am experiencing as it is openly without feeling ashamed of myself that I am having problems (P0123).<br>Confide (P071)<br>I can tell what's bothering me (P098).<br>I could talk about a lot of things, thanks to the description of this box! (P0106).
	Clear instruction and language	The instructions are easy to understand and clear (P0108).<br>The instructions are in the form of pointers and are clear (P0148).<br>Clear instruction and questions (P0128).<br>The language and the sequence are easy to follow (P0151).
	Others	Good material. A very modern and practical way of presenting the material (P023).<br>There is a voice recording that is a bit relaxing (P0135).<br>What I like about it is because it directs me to write. Remind me to write again (P061).<br>I like the writing, it is interesting (P044).<br>Had help me make specific schedule on the things I want to do…(P099).
**What did you not like about the sessions?**		
	Repeated questions	Repetition of questions (P0126).<br>Too many questions to answer (P0128).<br>The questions are difficult to answer because they are almost the same (P041).
	Technical problems	Maybe this system seems troublesome (P0106).<br>I don't know, I'm confused because I'm not a techno savvy, so somebody has to explain it to me many times for me to understand (P0135).<br>Not knowing the function of the '+' sign and how to use it (P0148).<br>The layout of the material presentation is less focused, I'm confused which one to read (P088).

	Confusion when doing exercise	Make a positive plan for the following week, because you don't know what to do (P0113).<br>The given example sometimes helps. But I think there is one step where I feel confused describing what my purpose is, I was tempted to follow the example (P0144).<br>I do not understand what to do with the stress analysis (P033).<br>I was still confused with the exercises that had to be done, the instructions were clear but I still don't understand how to do it (P044).<br>I was a little bit confused in doing exercise on defining stress… (P071).
**How could you benefit more from it?**		
	Real time communication with professional or eCoach	It would be more useful if there was a chat facility with the experts… (P0127).<br>I was struggling when I had panic attack and I became hysterical because I was so anxious but I could not contact the professional (P085).<br>There were mentors whom I can talk to (P085).<br>I want to have a question and answer session with professional (P071)
	Provide downloadable material	I hope there will be a PDF file that I can download from the results of what I filled in and hopefully I can print out the file so that I can read it again later and become my guidance in doing activities that are part of this training (P0123).<br>Printable positive activity plans (P033)<br>Provide material that can be saved such as pictures, ppt or notes so that they can be studied again later (P059).
	Simplify and concise	Make it more concise (P0104)<br>Maybe it can be made simpler and eye catching (P0106)<br>Shorten the illustration (P0128).<br>Simplify and make interesting layout (P088).
	Add music, more pictures, video	Use more songs to make me more relax (P043).<br>Play classical music when I am filling in the answer in each session (P099).<br>With more pictures (P032).<br>Adding videos will help (P0130).
	Better mobile interface	….the form of the scale on the cellphone confused me.. a better interface when I do the training via cellphone would be nice(P085).
**Other comment/ feedback**		
	Useful and helpful training/intervention	I really liked this training because I could share my problems that burden me and made me stress (P098).<br>Thank you for making this program (P0147).<br>It was such a pleasant and relaxing experience (P0135).

	Everything was very good and I felt the benefits of implementing these modules. Thank you very much for your help Rileks (P0147).<br>This intervention is very useful (P054).<br>Thank you for guiding me up to this stage. I am grateful since it helped a lot... Keep up the good work! (P0144).<br>…thank you for giving this training to me (P085)
